# Frequency and duration of physical activity bouts in school-aged children: A comparison within and between days

**DOI:** 10.1016/j.pmedr.2016.10.007

**Published:** 2016-10-26

**Authors:** Hannah L Brooke, Andrew J Atkin, Kirsten Corder, Soren Brage, Esther MF van Sluijs

**Affiliations:** MRC Epidemiology Unit & Centre for Diet and Activity Research (CEDAR), University of Cambridge, School of Clinical Medicine, Institute of Metabolic Science, Cambridge, UK

**Keywords:** Physical activity, Accelerometry, Patterns, Bouts, Time-segments, Intervention design, Children, Adolescents

## Abstract

Understanding how physical activity (PA) patterns vary within and between days may guide PA promotion in young people. We aimed to 1) describe and compare the frequency (bouts/day) and duration (min/bout) of bouts of moderate-to-vigorous intensity PA (MVPA) on weekdays vs. weekends and in-school vs. out-of-school, and 2) assess associations of bout frequency and duration in these time-segments with overall PA. We used cross-sectional accelerometer data from 2737 children (aged 6–19 years) in the United States National Health and Nutrition Examination Survey (NHANES) 2003–2006. A bout was defined as MVPA (≥ 2000 counts per minute [cpm]) lasting ≥ 3 min. Adjusted Wald tests were used to assess differences in bout characteristics between time-segments. Linear regression was used to examine the association of time-segment specific bout characteristics with daily minutes of MVPA and PA volume (average cpm). Bout frequency was higher on weekdays than weekends (median [IQR] 4.3 [2.2–7.2] vs. 3.0 [1.0–6.5] bouts/day, p < 0.001); however, bout duration did not differ (4.7 [4.0–5.7] vs. 4.5 [3.7–5.8] min/bout, p = 0.33). More bouts were accumulated out-of-school compared with in-school (2.2 [1.0–4.0] vs. 1.8 [0.8–3.2] bouts/day, p < 0.001), but bout duration was similar (4.7 [3.8–5.8] vs. 4.5 [3.8–5.7] min/bout, p = 0.158). For all time-segments, the frequency and duration of bouts of MVPA were independently and positively associated with overall MVPA and PA volume. In conclusion, the characteristics of children's PA vary within and between days; accounting for this in intervention design may improve future interventions. However, increasing bout frequency or duration in any time-segment may be beneficial for overall PA.

## Highlights

• MVPA accumulated on weekend days was more sporadic than MVPA accumulated on weekdays.
• PA patterns differed more between week vs. weekend days than in- vs. out-of-school.
• Variation in PA patterns across the week should be considered in intervention design.
• Bout frequency/duration had positive associations with overall PA in all time-periods.
• Increasing bout frequency/duration in any time-segment may help increase overall PA.

## 1 Introduction

Physical inactivity has been identified as one of the biggest challenges to public health in the 21st century (Blair, 2009). Promoting physical activity in childhood and encouraging the maintenance of physical activity in adolescence can help address this challenge (Blair, 2009, Department of Health, 2011, Janssen and Leblanc, 2010). However, physical activity interventions in young people are challenging and have had limited success (Metcalf et al., 2012). There is some evidence supporting the notion of tailoring physical activity interventions towards specific periods of time. For example, we have shown that it may be advantageous to target physical activity interventions at weekends and out-of-school hours on weekdays, due to the relatively large declines in activity observed during these periods between ages 10 and 14 years (Brooke et al., 2014). Although levels of physical activity differ across the week, we know little about the characteristics of physical activity in different time-segments (for example, weekdays, weekends, in-school and out-of-school), such as the types of activity performed or the frequency and duration of physical activity bouts. Differences in these characteristics between time-segments may help to explain differences in overall physical activity. Further understanding of the characteristics of physical activity, and how these characteristics vary between settings and time-segments, may help inform intervention design.

Physical activity bouts are periods of sustained activity of a specified minimum duration and intensity. In the UK, government recommendations suggest that adults should accumulate moderate-to-vigorous intensity physical activity (MVPA) in bouts of at least 10 min (Department of Health, 2011), but there is no guideline relating to bouts of activity for children. Despite this, accumulating physical activity in bouts may confer health benefits beyond that of physical activity accumulated more sporadically. For example, it has been shown that, independent of the overall volume of MVPA, young people in the highest quartile for bouts of MVPA were less likely to be overweight than those in the lowest quartile (Mark & Janssen, 2009). As such, understanding ways to promote bouts of activity or combine shorter bouts into longer ones may be important.

Two studies have explored frequency and duration of physical activity bouts for specific time-segments in children, indicating differences between periods of the week (Rowlands et al., 2008, McManus et al., 2011). One study suggested that lower physical activity on weekends may be due, in part, to lower frequency of higher intensity activity bouts on weekends (Rowlands et al., 2008). Another showed that the number and duration of bouts of physical activity were lower on weekends than in-school (McManus et al., 2011). However, these studies were conducted in small non-representative samples of young children and the analyses have not been replicated. Moreover, associations with overall activity have not been explored in young people, so it is unclear whether it would be relevant to target these characteristics in physical activity interventions.

We therefore aimed to 1) describe and compare the frequency and duration of bouts of MVPA on weekdays vs. weekend days and in-school vs. out-of-school, and 2) assess the associations of bout frequency and duration on weekdays, weekend days, in-school and out-of-school with overall physical activity in school-aged children.

## 2 Methods

### 2.1 Study outline

Data from the National Health and Nutrition Examination Survey (NHANES) were used for this study. The survey methodology is described in detail on the NHANES website (http://www.cdc.gov/nchs/nhanes.htm). Briefly, each year a representative sample (approximately 6000 individuals) of the United States (US) population are interviewed in their home and are then invited to attend a physical examination. In addition to the physical examination, from January 2003 until December 2006 participants aged 6 years and over were asked to wear an accelerometer for seven days to measure physical activity.

The NHANES protocol received National Center for Health Statistics (NCHS) Research Ethics Review Board approval. Participants aged 18 years and over provided written informed consent to participate. A parent or guardian provided written informed consent for individuals aged < 18 years to participate and assent was obtained from the participant. All data used in these analyses were fully anonymised and freely available to download from the NHANES website (http://www.cdc.gov/nchs/nhanes.htm).

### 2.2 Sample

To select a representative sample of the US population, NHANES uses a four-stage probability sampling design. The current analyses focus on children aged 6–19 years, who attended the physical examination between January 2003 and December 2006 (n = 6005). For inclusion in the analysis participants were required to be enrolled in primary or secondary education and provide a minimum of three valid days of accelerometer data including at least one weekend day (n = 2848) (Mattocks et al., 2008). We excluded 111 children without complete data for all covariates included in analyses (age, sex, body mass index [BMI], parental education, and ethnicity); this left 2737 children to be included in the final analytical sample.

### 2.3 Accelerometry

Participants were asked to wear an ActiGraph accelerometer (Model 7164, ActiGraph LCC, Ft. Walton Beach, FL) on a belt on their right hip for seven consecutive days. They were instructed to remove the monitor before sleeping, bathing, showering and swimming. Uniaxial accelerometers are commonly used to measure physical activity in young people (Cain et al., 2013) and they explain a moderate proportion (33 to 69%) of the variance in physical activity energy expenditure derived from doubly labelled water (Plasqui and Westerterp, 2007).

The accelerometers recorded movement in one-minute epochs from the day after they were fitted. Data are expressed in counts per minute (cpm) according to a proprietary algorithm employing frequency-based filtering (Brage et al., 2003). Counts before 6 am and after 11 pm each day, and strings of zero counts lasting 90 min or more, were removed from participants' data files. It was considered that the accelerometer was not worn during these periods (Cain et al., 2013). At least 600 min of data were required for a day to be considered valid.

### 2.4 Physical activity variables

• Physical activity volume (PA volume): total counts divided by total monitoring time each day.
• MVPA: time (minutes) spent at ≥ 2000 cpm (approximately equivalent to at least brisk walking in adolescents (Ekelund et al., 2003)).
• A bout: a period of semi-continuous MVPA lasting three or more minutes, allowing a one-epoch interruption, as recommended in previous literature (Masse et al., 2005), i.e. any second epoch below 2000 cpm ended the bout. Although previous studies have defined a bout as a minimum of 5 or 10 min of continuous activity, the three-minute cut-off was chosen in order for the definition to be behaviourally appropriate for the youngest children in the sample (Guvenc et al., 2013, Baquet et al., 2007).
• Bout frequency: total number of bouts performed on valid days divided by the number of valid days.
• Bout duration: total number of minutes of MVPA accumulated in bouts on valid days, divided by the total number of bouts on valid days. NB. This was only calculated for those children who performed at least one activity bout.

In addition we derived:

• Absolute frequency of, and time (minutes) in, MVPA accumulated as 1) non-bout activity (i.e. < 3 min in duration), 2) bouts of 3 to 5 min, 3) bouts of 6 to 10 min, and 4) bouts > 10 min in duration.
• Relative frequency of, and time in, MVPA accumulated as non-bout activity and bouts of different durations (described above) i.e. as proportions of all occurrences of MVPA and all minutes of MVPA, respectively.

Physical activity variables were calculated separately for weekdays, weekend days, in-school (0800–1500, weekdays) and out-of-school (0600–0800 and 1500–2300, weekdays). The definitions of in-school and out-of-school were based on a previous study of children in the US education system (Long et al., 2013).

### 2.5 Measurement of covariates

Participants' age, sex, ethnicity and parent or guardian education level were determined at the home interview. For participants aged < 16 years, interviews were conducted with a parent or guardian as a proxy for the participant. Ethnicity was categorised as “non-Hispanic white”, “non-Hispanic black”, “Mexican American”, and “Other” (which included Multi-Racial persons and other Hispanic persons). Parent or guardian education level was categorised as “Less than high school graduate”, “High school graduate or equivalent”, “Some college or foundation degree”, “College graduate or higher”.

At the physical examination participants were dressed in light clothing and standard procedures were used to measure their height and weight (http://www.cdc.gov/nchs/data/nhanes/nhanes_03_04/BM.pdf). BMI was calculated as weight in kg divided by height in m^2^. Individuals were categorised using sex- and age- dependent cut-points into “Normal weight or underweight”, “Overweight”, and “Obese” (Cole et al., 2000).

### 2.6 Statistical analyses

#### 2.6.1 Accounting for survey design

To conduct appropriate design-based analyses, sample weights, clustering, and stratification, were accounted for. Data from the 2003–2004 and 2005–2006 were combined by multiplying the sample weights by 0.5. To account for individuals excluded from the analytical sample the weightings were recalculated within age, sex and ethnicity groupings, based on a Statistical Analysis Systems (SAS) program provided by the National Cancer Institute (National Cancer Institute, 2007). This has previously been shown to generate a representative sample (Troiano et al., 2008). Survey commands in Stata 13 (StataCorp. 2013. Stata Statistical Software: Release 13. College Station, TX: USA) were used in all analyses to ensure that the sampling design was correctly accounted for.

#### 2.6.2 Demographic and anthropometric characteristics

Differences in demographic and anthropometric characteristics based on participants who were included and excluded from the analytical sample were tested using linear regression (continuous outcomes) or multinomial logistic regression (categorical outcomes).

#### 2.6.3 Differences in characteristics of physical activity between time-segments

Due to the skewed distributions of the raw variables, we derived the difference in MVPA, TPA, bout frequency and bout duration between time-segments (weekdays minus weekend days and in-school minus out-of-school; normally distributed variables). We then used the adjusted Wald test to examine whether differences between time-segments were equal to zero. This was the most appropriate test because multilevel models are not supported in Stata 13 when using survey commands. Therefore, repeated observations at the individual level (i.e. weekdays and weekend days/in-school and out-of-school) could not be correctly accounted for using linear regression, at the same time as accounting for the survey design. The same methods were used to assess differences between time-segments in both the absolute and the relative frequency (as defined above) of, and time in, MVPA accumulated in non-bout activity and bouts of 3–5, 6–10 and > 10 min in duration. Preliminary analyses indicated no evidence of sex by time-segment interaction; as such, analyses were not stratified by sex.

Children accumulating physical activity in non-bout activity can achieve high levels of overall physical activity. As such, it is important for future physical activity guidelines and interventions to determine whether bout frequency and bout duration per se are important for overall levels of physical activity. We tested the association of bout frequency and bout duration, as mutually adjusted independent variables, with overall PA volume and overall MVPA using linear regression. These analyses were run for weekdays, weekend days, in-school and out-of-school in separate models. Age, sex, age-standardized BMI, ethnicity, and parental education were included as potential confounders.

### 2.7 Sensitivity analyses

Periods of vacation are likely to disrupt the distinction between time-segments. As such, we conducted a sensitivity analysis excluding all children who were reported to be on vacation at the home interview. Physical activity was typically measured several weeks after the home interview, so misclassification in the sensitivity analysis is possible. However, this was the only variable available indicating whether or not the participant may have been on vacation from school at the point of data collection.

## 3 Results

### 3.1 Sample characteristics

Demographic and anthropometric characteristics based on children included in the analytical sample (n = 2737) were similar to the characteristics based on those excluded (n = 3268) for sex, age, height, weight, BMI, weight status and ethnicity (p-values all > 0.16). Parental educational level based on children included in the analytical sample was higher than the parental educational level based on those who were excluded (23% vs. 19% of parents/guardians achieved college education or higher, p = 0.011). The sample weighted mean age based on those children included in the analytical sample was 12.6 years; the majority of children were normal weight (67.1%) and non-Hispanic white (61.8%) (Table 1).

### 3.2 Differences in characteristics of physical activity between time-segments

On weekdays, compared with weekend days, more MVPA was accumulated, and bout frequency was higher. PA volume and bout duration did not differ between these time-segments (Table 2). Out-of-school, more MVPA was accumulated, PA volume was higher, and there were more bouts, than in-school (Table 2). However, bout duration was similar in-school and out-of-school.

**Fig. 1:**
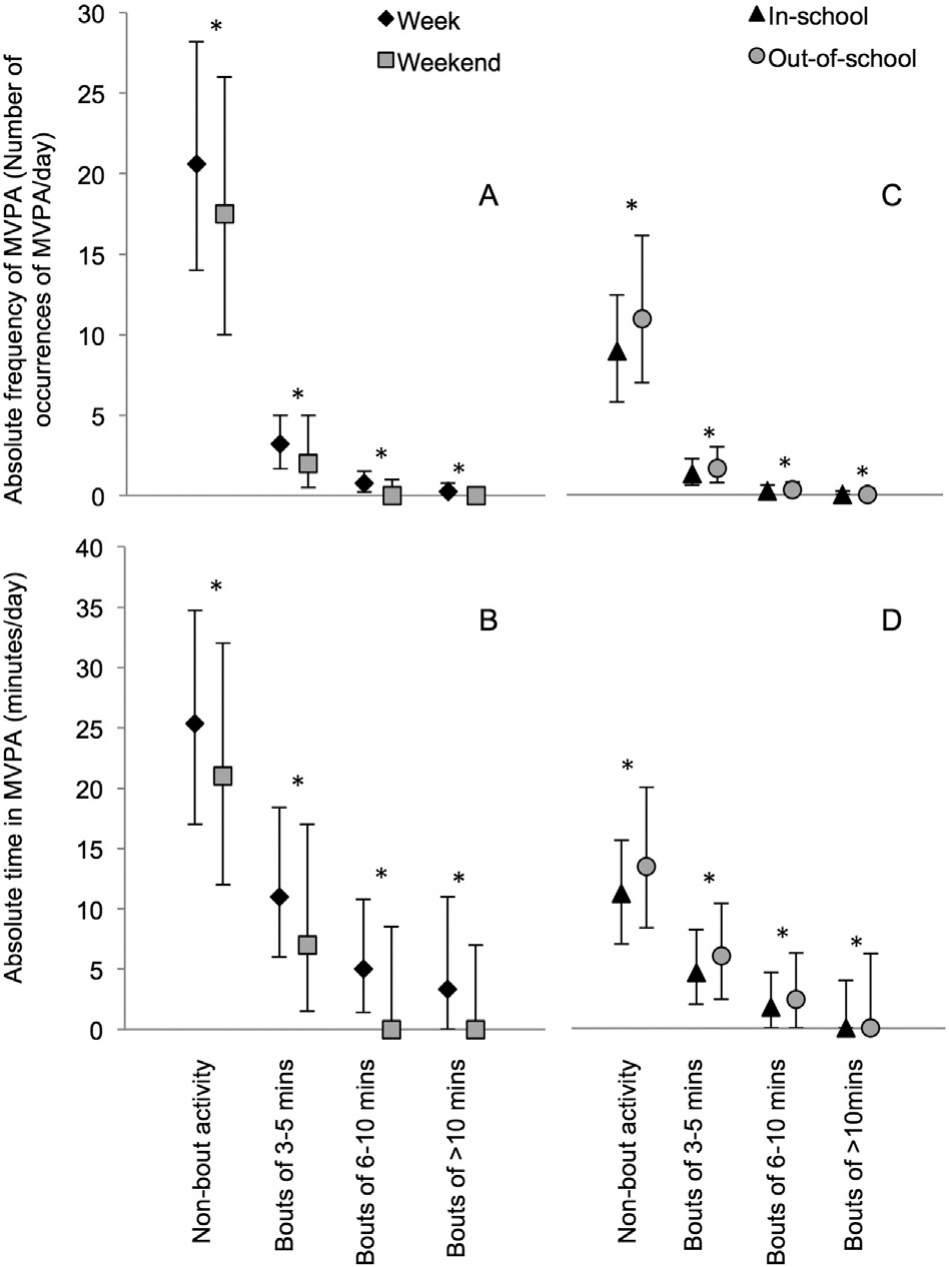
Absolute frequency of, and time in, MVPA accumulated in non-bout activity and bouts of different durations (median and interquartile range). MVPA, moderate-to-vigorous intensity physical activity. *p-Values < 0.05; derived from adjusted Wald tests assessing whether differences between time-segments (weekdays minus weekend days and in-school minus out-of-school; normally distributed variables) were equal to zero. Based on data from children in the United States National Health and Nutrition Examination Survey (NHANES) 2003–2006.

**Fig. 2:**
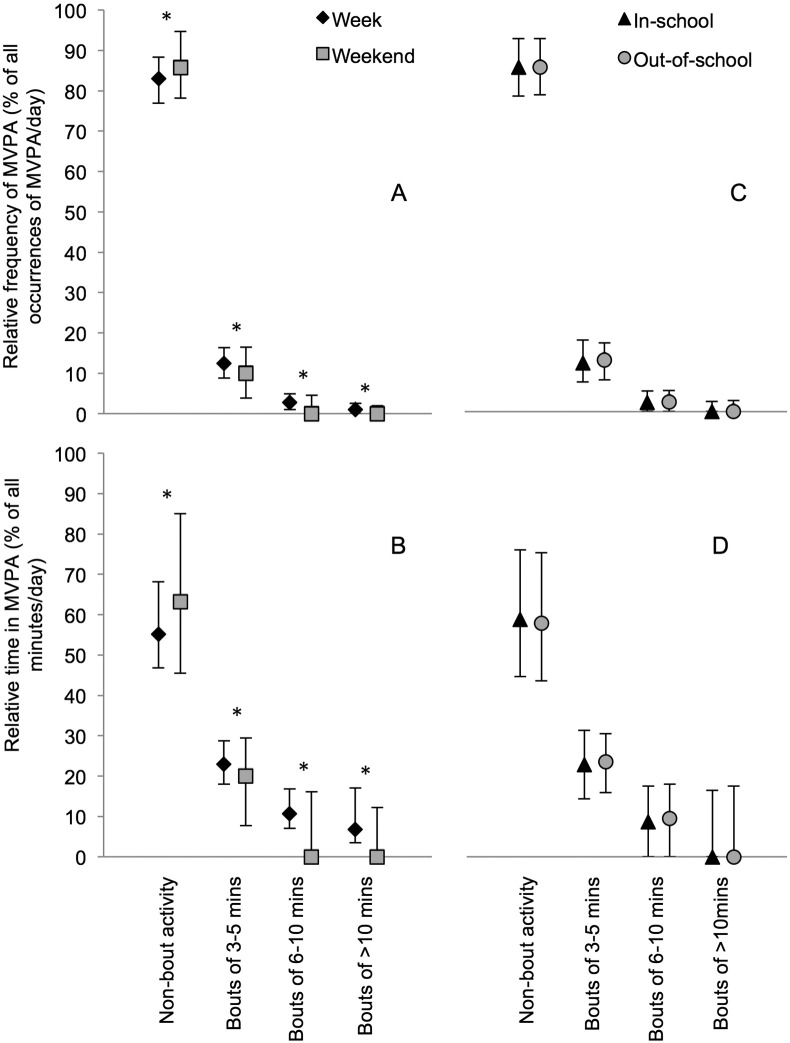
Relative frequency of, and time in, MVPA accumulated in non-bout activity and bouts of different durations (median and interquartile range) i.e. as proportions of all occurrences of MVPA and all minutes of MVPA, respectively. MVPA, moderate-to-vigorous intensity physical activity. *p-Values < 0.05; derived from adjusted Wald tests assessing whether differences between time-segments (weekdays minus weekend days and in-school minus out-of-school; normally distributed variables) were equal to zero. Based on data from children in the United States National Health and Nutrition Examination Survey (NHANES) 2003–2006. Based on children who achieved at least 1 min of MVPA in that time-segment (weekdays, n = 2736; weekend days, n = 2719; in-school, n = 2727; out-of-school, n = 2733).

The absolute frequency of, and time in, MVPA accumulated in non-bout activity and bouts of 3–5, 6–10 and > 10 min was higher on weekdays than weekend days (Fig. 1A and B), and out-of-school compared with in-school (Fig. 1C and D). The relative frequency of, and time in, MVPA accumulated in bouts was also higher on weekdays than weekend days (Fig. 2A and B). In contrast, the relative frequency of, and time in, MVPA accumulated in non-bout activity was higher on weekend days than on weekdays. In addition, there were no differences between in-school and out-of-school (non-)bout characteristics when frequency of, and time in, MVPA were considered as a proportion of all occurrences or minutes of MVPA (i.e. relative, see Fig. 2C and D).

### 3.3 Associations of time-segment specific physical activity characteristics with overall physical activity

As can be seen from Fig. 2, around 85% of all occurrences of MVPA and around 60% of all minutes of MVPA were accumulated in ‘non-bout’ activity. As such, it was important to examine whether children performing more bouts of activity were more active than other children overall or whether other children were accumulating just as much MVPA and overall volume of PA in non-bout activity. In all time-segments, bout frequency and bout duration had small to moderate sized independent positive associations with overall PA volume and MVPA (Table 3). For bout frequency, the strongest associations were for the out-of-school time period (β Coef. [95% CI]: PA volume, 51.5 [48.1, 54.49] cpm; and MVPA, 9.0 [8.5, 9.5] min). For bout duration, the strongest associations were for weekdays (β Coef. [95% CI]: PA volume, 15.2 [11.2, 19.2] cpm; and MVPA, 2.1 [1.7, 2.5] min). Age, sex, age-standardized BMI, ethnicity, and parental education were included as potential confounders in these analyses. Age, sex, and age-standardized BMI were the most consistent covariates contributing to models described above (Supplementary tables 1 and 2).

In sensitivity analyses excluding individuals on vacation from school at the interview stage of data collection, summary statistics varied slightly from the main analysis. However, the overall conclusions from each analysis reflected the main results.

## 4 Discussion

The frequency and duration of bouts of MVPA differed between specific time periods of the day and week. In all time-segments, bout frequency and duration showed independent small-to-moderate sized positive associations with overall physical activity. Findings highlight a novel approach that could be applied to enhance effectiveness of future interventions. Our findings suggest that if an intervention were to successfully increase either bout frequency or bout duration in any time-segment it may be beneficial for overall physical activity levels.

The results suggest that lower overall MVPA on weekend days (compared to weekdays) may be due to children participating in fewer bouts of physical activity rather than in bouts of shorter duration. This echoes previous findings that frequency of activity bouts is lower on weekends than on weekdays (Rowlands et al., 2008). For interventions targeting weekend physical activity, it may therefore be appropriate to focus on bout frequency (i.e. incorporating more physical activity sessions into the day) rather than bout duration (i.e. requiring young people to be active for prolonged periods of time), as there may be greater scope to affect behaviour using this approach. Bout frequency may be increased in interventions that aim to break up sedentary time with bouts of physical activity (Manios et al., 2014). Moreover, interventions that use goal setting methods to promote physical activity may be more effective if the goals focused on frequency of MVPA bouts rather than bout duration (Rhodes et al., 2010).

The relative frequency of, and time in, MVPA accumulated in non-bout activity was higher on weekend days than on weekdays. This suggests that MVPA accumulated on weekend days may be more sporadic than MVPA accumulated on weekdays. Differences in bout characteristics between weekdays and weekends persisted when the frequency and minutes of MVPA accumulated in bouts were considered as proportions of all episodes and minutes of MVPA. In contrast, there were no differences between in-school and out-of-school time periods when bout characteristics were expressed as a relative measure. The observed differences between bout characteristics in-school and out-of-school were therefore largely due to differences in overall physical activity accumulated in these time-segments. This suggests that the characteristics of physical activity differ more between weekdays and weekends than between in-school and out-of-school time periods. As such, the characteristics of physical activity may be particularly important to take into account when developing interventions targeting weekend days.

As previously shown (Cliff et al., 2014), bouts of longer duration were uncommon. However, if the minutes of MVPA accumulated are also considered, it becomes clear that longer duration bouts, particularly on weekdays, do contribute to young peoples' overall physical activity levels, despite their low frequency. As such, developing interventions that encourage longer bouts of MVPA could be effective because there is scope to increase the frequency of bouts of this length. For example, young people may be encouraged to extend short bouts of physical activity into longer bouts. A small increase in the number of medium-to-long duration bouts of MVPA could have a large impact on the overall amount of MVPA time accumulated. However, it is also important to consider that the infrequency of longer duration bouts of MVPA may be because longer bouts are not behaviourally (or, in younger children, developmentally) appropriate or appealing for this population. However, it may be appropriate to help adolescents become accustomed to bouts of longer duration, given that MVPA should be accumulated in bouts of at least 10 min in adulthood according to current health recommendations (Department of Health, 2011).

One of the strengths of this study was using data from a large sample representative of the US population. As such, selection bias influencing internal or external validity is thought to be minimal. Nonetheless, the generalizability of the results to populations outside of the US should still be established. Further strengths include objective ƒmeasurement of physical activity, within-person analysis of time-segment specific differences in the characteristics of physical activity, and analytical techniques that correctly accounted for the study's sampling design. The study also has some limitations. For example, the accelerometry protocol used in these waves of NHANES does not capture physical activity during water-based activities and is limited in its representation of some activities, such as cycling (Corder et al., 2007). This may have influenced the results if these activities were more likely during some time periods (e.g. weekends or out-of-school) than others. Moreover, since physical activity was averaged within 60-second epochs we cannot be sure that activity was truly continuous within each epoch. Future studies should, if possible, use physical activity monitors set to a shorter epoch length to gather a greater level of detail about the patterns of children's physical activity. School start and end times may differ between schools and between states, which may have led to some misclassification of in-school time as out-of-school and vice versa. This is likely to have made physical activity in different periods of time more similar, thus reducing the effect sizes of differences between time-segments. Similarly, some children may have been on vacation during the physical activity data collection period. Data were not available to test the potential influence of including summer months in the analyses. However, the main conclusions were not different when individuals on vacation at the interview stage of data collection were excluded from analyses. Furthermore, including children on vacation is likely to result in conservative effect estimates, due to misclassification of out-of-school time as in-school time. This greater measurement error would be expected to reduce the power of the analyses. Such misclassification may also have influenced the accuracy of the physical activity estimates (e.g. Table 2). However, given the large sample size, and the relatively small number of individuals on vacation during data collection, this is unlikely to have substantially impacted the study findings.

We present bout duration and frequency for a wide age range of children and show differences in characteristics between time-segments. Examining correlates of physical activity characteristics was outside the scope of the current manuscript, but in view of the reduction in physical activity that accompanies the transition from childhood to adolescence, future studies should examine how the characteristics of children's physical activity, such as bout frequency and duration, in different time-segments vary with age. Finally, bouts of physical activity can be defined in different ways. In this study we defined a bout based on semi-continuous MVPA (i.e. allowing a one epoch interruption in MVPA). As such, we will have a higher mean bout duration and a higher number of longer bouts than would have occurred had we defined a bout based on continuous MVPA (i.e. no interruptions permitted). This is likely to influence bouts recorded in all time-segments to a similar extent, so the overall results would be unlikely to change if a different definition of a bout had been used.

## 5 Conclusions

More MVPA was accumulated, and bout frequency was higher, on weekdays compared with weekend days. MVPA accumulated on weekend days was more sporadic than MVPA accumulated on weekdays. Moreover, the characteristics of physical activity differed more between weekdays and weekends than between in-school and out-of-school time periods. Accounting for these differences in physical activity patterns between time-segments may contribute to the design of future interventions. A focus upon increasing bout frequency may be worthwhile in physical activity interventions targeting weekends. Bout frequency and duration were independently positively associated with overall physical activity in all time-segments. An intervention increasing either of these characteristics in any time-segment may therefore help increase overall activity.

## Conflicts of interest

None.

## Figures and Tables

**Table 1:** Demographic and anthropometric characteristics based on children from the United States National Health and Nutrition Examination Survey (NHANES) 2003–2006 included in the analytical sample (n = 2737).

	Mean or percent (s.e.)	
Sex		
Boys	51.2	(1.3)
Girls	48.8	(1.3)
Age (years)	12.6	(0.1)
Height (cm)	152.3	(0.6)
Weight (kg)	50.3	(0.7)
BMI (kg/m^2^)	20.9	(0.2)

Weight status		
Normal weight	67.1	(1.7)
Overweight	20.3	(1.2)
Obese	12.7	(1.0)

Ethnicity		
Non-Hispanic white	61.8	(2.9)
Non-Hispanic black	14.6	(1.8)
Mexican American	12.2	(1.5)
Other	11.3	(1.3)

Parental Education		
Less than high school graduate	17.5	(1.3)
High school graduate or equivalent	25.7	(2.0)
Some college or equivalent	34.3	(1.4)
College graduate or above	22.5	(1.8)

Mean or percent (standard error [s.e.]) weighted to account for the survey design characteristics and inclusion in the analytical sample.

BMI, Body mass index.

**Table 2:** MVPA, PA volume, bout frequency and bout duration for weekdays vs. weekend days and in-school vs. out-of-school.

	Weekdays		Weekend days		Week vs. weekend
	Median	(IQR)	Median	(IQR)	p-Value
MVPA (min)	47.5	(29.5, 72.2)	35.5	(16.5, 67.0)	< 0.001
PA volume (cpm)	465.0	(336.0, 616.6)	446.7	(292.2, 639.5)	0.631
Bout frequency (n/day)	4.3	(2.2, 7.2)	3.0	(1.0, 6.5)	< 0.001
Bout duration (min/bout)^a^	4.7	(4.0, 5.7)	4.5	(3.7, 5.8)	0.330

	In-school		Out-of-school		In-school vs. out-of-school
	Median	(IQR)	Median	(IQR)	p-Value
MVPA (min)	20.3	(11.8, 32.0)	26.0	(14.0, 41.4)	< 0.001
PA volume (cpm)	416.7	(306.7, 566.9)	485.4	(336.0, 666.4)	< 0.001
Bout frequency (n/day)	1.8	(0.8, 3.2)	2.2	(1.0, 4.0)	< 0.001
Bout duration (min/bout)^a^	4.5	(3.8, 5.7)	4.7	(3.8, 5.8)	0.158

MVPA, moderate-to-vigorous intensity physical activity; PA volume, physical activity volume; cpm, counts per minute; IQR, inter quartile range.

Bout frequency and duration were positively skewed and therefore physical activity data are presented as median and interquartile range.

p-Values are derived from adjusted Wald tests assessing whether differences between time-segments (weekdays minus weekend days and in-school minus out-of-school; normally distributed variables) were equal to zero.

Based on data from children in the United States National Health and Nutrition Examination Survey (NHANES) 2003–2006.

a Bout duration was only calculated for those children who performed at least one activity bout (weekdays, n = 2674; weekend days, n = 2171; in-school, n = 2513; out-of-school, n = 2582).

**Table 3:** Associations of time-segment specific mean bout frequency and duration with overall PA volume and MVPA.

	Time-segment	Bout characteristic	β Coef.	(95% CI)	p-Value
PA volume (cpm)	Weekday	Frequency	37.3	(35.1, 39.6)	< 0.001
		Duration	15.2	(11.2, 19.2)	< 0.001
	Weekend	Frequency	22.0	(19.1, 25.0)	< 0.001
		Duration	10.4	(4.3, 16.4)	0.002
	In-school	Frequency	46.6	(40.6, 52.6)	< 0.001
		Duration	8.4	(5.4, 11.4)	< 0.001
	Out-of-school	Frequency	51.5	(48.1, 54.9)	< 0.001
		Duration	10.8	(6.8, 14.8)	< 0.001
MVPA (min)	Weekday	Frequency	6.8	(6.5, 7.1)	< 0.001
		Duration	2.1	(1.7, 2.5)	< 0.001
	Weekend	Frequency	3.9	(3.6, 4.2)	< 0.001
		Duration	1.1	(0.4, 1.8)	0.004
	In-school	Frequency	8.9	(8.0, 9.8)	< 0.001
		Duration	1.1	(0.6, 1.6)	< 0.001
	Out-of-school	Frequency	9.0	(8.5, 9.5)	< 0.001
		Duration	1.7	(1.2, 2.1)	< 0.001

PA volume, physical activity volume; cpm, counts per minute; MVPA, moderate to vigorous intensity physical activity; β Coef., beta-coefficient (unstandardized); 95% CI, 95% confidence interval.

Linear regression models adjusted for age, sex, age-standardized BMI, ethnicity, and parental education (beta-coefficients and 95% confidence intervals for these covariates are presented in Supplementary tables 1 and 2), mutually adjusted for bout frequency and duration.

Based on data from children in the United States National Health and Nutrition Examination Survey (NHANES) 2003–2006.

Analyses only included those children who performed at least one activity bout (weekdays, n = 2674; weekend days, n = 2171; in-school, n = 2513; out-of-school, n = 2582).

## Appendix A Supplementary data

Supplementary tables. Image 1
