# Supplementary material for: Frequency and duration of physical activity bouts in school-aged children: A comparison within and between days
Source: Prev Med Rep. 2016 Oct 26;4:585–90. doi: 10.1016/j.pmedr.2016.10.007 (PMC5107648; doi:10.1016/j.pmedr.2016.10.007)
Supplement: Supplementary file 1 — Supplementary tables. [file mmc1.pdf]

Supplementary table 1. Full models used to examining associations of time-segment specific mean bout frequency and duration with overall PA volume

Time-segment	Weekday			Weekend			In-school			Out-of-school		
	β Coef.	95% CI	P-value	β Coef.	95% CI	P-value	β Coef.	95% CI	P-value	β Coef.	95% CI	P-value
**Bout characteristic**												
Frequency	37.3	(35.1, 39.6)	<0.001*	22.0	(19.1, 25.0)	<0.001*	46.6	(40.6, 52.6)	<0.001*	51.5	(48.1, 54.9)	<0.001*
Duration	15.2	(11.2, 19.2)	<0.001*	10.4	(4.3, 16.4)	0.002*	8.4	(5.4, 11.4)	<0.001*	10.8	(6.8, 14.8)	<0.001*
**Sex**												
Boys	Ref			Ref			Ref			Ref		
Girls	-6.2	(-18.0, 5.7)	0.296	-37.9	(-53.3, -22.5)	<0.001*	-42.9	(-59.4, -26.4)	<0.001*	-27.8	(-39.3, -16.2)	<0.001*
**Age**	-21.2	(-23.2, -19.2)	<0.001*	-20.8	(-24.1, -17.5)	<0.001*	-25.6	(-28.2, -22.9)	<0.001*	-21.9	(-23.9, -19.8)	<0.001*
**Ethnicity**												
Non-Hispanic White	Ref			Ref			Ref			Ref		
Non-Hispanic black	-16.9	(-35.0, 1.2)	0.066	15.9	(-4.6, 36.4)	0.124	19.1	(-5.9, 44.1)	0.129	-23.3	(-42.6, -4.0)	0.020*
Mexican American	-36.9	(-52.2, -21.5)	<0.001*	-3.8	(-23.8, 16.3)	0.704	-34.5	(-54.3, -14.6)	0.001*	-20.4	(-37.7, -3.1)	0.022*
Other	-29.0	(-48.6, -9.4)	0.005*	-15.6	(-39.5, 8.3)	0.193	-30.6	(-57.6, -3.6)	0.028*	-25.7	(-47.9, -3.6)	0.024*
**Age-standardised BMI**	-1.2	(-2.5, 0.1)	0.062	-3.2	(-4.5, -2.0)	<0.001*	-4.2	(-6.0, -2.4)	<0.001*	-1.1	(-2.3, 0.1)	0.061
**Parental education**												
Less than high school	Ref			Ref			Ref			Ref		
High school	8.0	(-14.5, 30.6)	0.474	7.1	(-14.5, 28.7)	0.508	6.3	(-17.3, 29.9)	0.591	14.1	(-8.9, 37.2)	0.220
Some college	-0.5	(-22.7, 21.7)	0.963	2.6	(-16.6, 21.7)	0.786	-2.5	(-25.4, 20.3)	0.822	-2.8	(-22.9, 17.3)	0.776
College graduate	-19.2	(-36.2, -2.2)	0.028*	-11.1	(-38.4, 16.1)	0.411	-20.3	(-41.8, 1.1)	0.062	-9.0	(-28.3, 10.3)	0.349

PA volume, physical activity volume; cpm, counts per minute; β Coef., beta-coefficient (unstandardized); 95% CI, 95% confidence interval; BMI, body mass index.

Linear regression models

Based on data from children in the United States National Health and Nutrition Examination Survey (NHANES) 2003-2006.

Analyses only included those children who performed at least one activity bout (weekdays, n=2674; weekend days, n=2171; in-school, n=2513; out-of-school, n=2582)

*Statistically significant contribution in the regression analysis

Supplementary table 2. Full models used to examining associations of time-segment specific mean bout frequency and duration with overall MVPA

Time-segment	Weekday			Weekend			In-school			Out-of-school		
	β Coef.	95% CI	P-value	β Coef.	95% CI	P-value	β Coef.	95% CI	P-value	β Coef.	95% CI	P-value
**Bout characteristic**												
Frequency	6.8	(6.5, 7.1)	<0.001*	3.9	(3.6, 4.2)	<0.001*	8.9	(8.0, 9.8)	<0.001*	9.0	(8.5, 9.5)	<0.001*
Duration	2.1	(1.7, 2.5)	<0.001*	1.1	(0.4, 1.8)	0.004*	1.1	(0.6, 1.6)	<0.001*	1.7	(1.2, 2.1)	<0.001*
**Sex**												
Boys	Ref			Ref			Ref			Ref		
Girls	-3.7	(-5.0, -2.3)	<0.001*	-10.7	(-13.0, -8.3)	<0.001*	-9.7	(-12.3, -7.2)	<0.001*	-8.0	(-9.8, -6.2)	<0.001*
**Age**	-1.4	(-1.7, -1.2)	<0.001*	-1.5	(-2.0, -1.1)	<0.001*	-2.2	(-2.6, -1.8)	<0.001*	-1.6	(-1.9, -1.3)	<0.001*
**Ethnicity**												
Non-Hispanic White	Ref			Ref			Ref			Ref		
Non-Hispanic black	0.1	(-1.9, 2.1)	0.956	6.3	(4.0, 8.7)	<0.001*	6.6	(3.3, 10.0)	<0.001*	-0.7	(-3.1, 1.7)	0.569
Mexican American	-2.6	(-4.0, -1.2)	0.001*	3.4	(0.5, 6.2)	0.022*	-2.5	(-5.4, 0.4)	0.091	0.5	(-1.7, 2.7)	0.663
Other	-2.2	(-4.4, -0.1)	0.044*	-0.3	(-3.3, 2.8)	0.862	-2.2	(-6.5, 2.2)	0.315	-1.6	(-4.4, 1.1)	0.239
**Age-standardised BMI**	-0.2	(-0.4, -0.1)	0.002*	-0.5	(-0.7, -0.3)	<0.001*	-0.8	(-1.0, -0.5)	<0.001*	-0.2	(-0.4, 0.0)	0.014*
**Parental education**												
Less than high school	Ref			Ref			Ref			Ref		
High school	0.6	(-1.2, 2.3)	0.525	0.4	(-2.5, 3.3)	0.796	-0.3	(-3.1, 2.5)	0.806	1.4	(-1.0, 3.9)	0.241
Some college	-0.2	(-2.1, 1.8)	0.868	0.4	(-2.0, 2.9)	0.725	-0.6	(-3.2, 2.0)	0.654	-0.6	(-2.9, 1.7)	0.597
College graduate	-0.6	(-2.4, 1.2)	0.499	1.4	(-3.1, 6.0)	0.528	-1.4	(-4.8, 2.0)	0.413	1.2	(-1.6, 4.0)	0.381

MVPA, moderate to vigorous intensity physical activity; β Coef., beta-coefficient (unstandardized); 95% CI, 95% confidence interval; BMI, body mass index.

Linear regression models

Based on data from children in the United States National Health and Nutrition Examination Survey (NHANES) 2003-2006.

Analyses only included those children who performed at least one activity bout (weekdays, n=2674; weekend days, n=2171; in-school, n=2513; out-of-school, n=2582)

*Statistically significant contribution in the regression analysis
